# A bovine model of rhizomelic chondrodysplasia punctata caused by a deep intronic splicing variant in the *GNPAT* gene

**DOI:** 10.1186/s12711-025-00969-z

**Published:** 2025-05-20

**Authors:** Arnaud Boulling, Julien Corbeau, Cécile Grohs, Anne Barbat, Jérémy Mortier, Sébastien Taussat, Vincent Plassard, Hélène Leclerc, Sébastien Fritz, Cyril Leymarie, Lorraine Bourgeois-Brunel, Alain Ducos, Raphaël Guatteo, Didier Boichard, Mekki Boussaha, Aurélien Capitan

**Affiliations:** 1 Université Paris Saclay, INRAE, AgroParisTech, GABI, 78350 Jouy-en-Josas, France; 2 Oniris, INRAE, BIOEPAR, 44300 Nantes, France; 3 Service d’imagerie Médicale, DEPEC, Ecole Nationale Vétérinaire d’Alfort, 94700 Maisons-Alfort, France; 4 ELIANCE, 75012 Paris, France; 5 OS Aubrac, 12000 Rodez, France; 6 GenPhySE, Université de Toulouse, INRAE, ENVT, 31326 Castanet Tolosan, France

## Abstract

**Background:**

Genetic defects that occur naturally in livestock species provide valuable models for investigating the molecular mechanisms underlying rare human diseases. Livestock breeds are subject to the regular emergence of recessive genetic defects due to genetic drift and recent inbreeding. At the same time, their large population sizes provide easy access to case and control individuals and to massive amounts of pedigree, genomic and phenotypic information recorded for management and selection purposes. In this study, we investigated a lethal form of recessive chondrodysplasia observed in 21 stillborn calves of the Aubrac beef cattle breed.

**Results:**

Detailed examinations of three affected calves revealed proximal limb shortening, epiphyseal calcific deposits, and other pathological signs consistent with human rhizomelic chondrodysplasia punctata, a rare peroxisomal disorder caused by recessive variants in one of five genes (*AGPS, FAR1*, *GNPAT*, *PEX5,* and *PEX7*). Using homozygosity mapping, whole genome sequencing of two affected individuals, and filtering for variants found in 1867 control genomes, we reduced the list of candidate variants to a single deep intronic substitution in *GNPAT* (NC_037355.1:g.4039268G > A on chromosome 28 of the ARS-UCD1.2 bovine genome assembly). For verification, we performed large-scale genotyping of this variant using a custom SNP array and found a perfect genotype–phenotype correlation in 21 cases and 26 of their parents, and a complete absence of homozygotes in 1195 unaffected Aubrac controls. The g.4039268A allele segregated at a frequency of 2.6% in this population and was absent in 375,535 additional individuals from 17 breeds. Then, using in vivo and in vitro analyses, we demonstrated that the derived allele activates cryptic splice sites within intron 11 resulting in abnormal transcripts. Finally, by mining the wealth of records available in the French bovine database, we also reported suggestive effects on juvenile mortality (and not just stillbirth) in homozygotes and on muscle development in heterozygotes, which merit further investigation.

**Conclusions:**

We report the first spontaneous large animal model of rhizomelic chondrodysplasia punctata and provide a diagnostic test to select against this defect in cattle. Our work also brings interesting insights into the molecular consequences of complete or partial GNPAT insufficiency in mammals.

**Supplementary Information:**

The online version contains supplementary material available at 10.1186/s12711-025-00969-z.

## Background

Over the past decade, high-throughput genotyping and next-generation sequencing have significantly advanced clinical research. Thousands of disease-causing variants have been identified in both humans and non-model species [1]. However, most genetic studies are biased by a tendency to focus on the exome because of the challenges of annotating non-coding regions of the genome. Additionally, whole-exome sequencing is often cheaper compared to whole-genome sequencing [2].

While there is growing evidence supporting the role of non-coding variations in disease pathogenesis [3], studying these variations in humans faces several limitations. Many disorders remain poorly understood primarily due to the small number of patients affected by rare genetic defects and the relatively high genetic variability of humans compared to laboratory and domestic animal species. This variability complicates the process of filtering variants without functional annotation.

Moreover, obtaining various tissues from living or deceased patients can be challenging due to health risks and ethical or religious concerns. These difficulties often hinder comprehensive clinical and pathological investigations and functional validation.

In this context, naturally occurring genetic defects in livestock species represent valuable models for studying the molecular mechanisms underlying rare human diseases. Farm animals are divided into numerous inbred populations or breeds that are prone to the regular emergence of recessive genetic defects [4]. Their large population sizes also facilitate access to both case and control individuals, along with extensive pedigree, genomic, and phenotypic information that has been recorded for management and selection purposes [5, 6].

From 2002 to 2020, the French National Observatory for Bovine Abnormalities (ONAB) received 21 reports of stillborn Aubrac calves with severe skeletal dysplasia, initially suspected to exhibit Bulldog Calf Syndrome (BDS). This syndrome is a congenital form of bovine chondrodysplasia that has been associated with harmful variants in the aggrecan (*ACAN*) and collagen type II alpha 1 chain (*COL2A1*) genes [5, 7–13]. Pedigree analysis and pathological examination indicated that this genetic defect follows a recessive inheritance pattern and is similar to human rhizomelic chondrodysplasia punctata (RCDP) [14]. RCDP is a recessive peroxisomal disease caused by variants in five genes: *AGPS*, *FAR1*, *GNPAT, PEX5,* and *PEX7,* which encode alkylglycerone phosphate synthase, fatty alcohol reductase 1, glycerone-phosphate O-acyltransferase, and the peroxisomal biogenesis factors 5 and 7, respectively [15–20].

This article describes how we have (i) identified a single deep intronic substitution in the *GNPAT* gene as a candidate causative variant for RCDP in Aubrac cattle, and (ii) characterised its molecular and clinical effects in both homozygous and heterozygous states, taking advantage of the abundant resources available in this species. In summary, we present the first large animal model of RCDP and a unique example of a deep intronic variant that leads to a genetic defect in livestock [21] to which has been assigned the corresponding causal variant (omia.variant:1804; https://www.omia.org/variant/omia.variant:1804/).

## Methods

### Animals

Over an 18-year period, 21 stillborn calves (8 males and 13 females) with a severe form of skeletal dysplasia were observed in 21 purebred Aubrac herds.

Seventeen owners of the affected calves recorded parental information, which was used to extract detailed pedigree records from the French national pedigree database. Veterinarians and artificial insemination (AI) technicians conducted a rough clinical description in the field and collected ear biopsies and photographs of all affected calves. Due to the rapid removal of carcasses by rendering companies, only three affected calves could be recovered for complete necropsy and pathological examination. Additionally, the body of an unaffected calf that died of natural causes at four days of age was also collected to serve as a control.

At the time of the study, biological samples for DNA extraction (including EDTA blood tubes or cryopreserved semen straws) were available for 15 dams and 11 sires of the affected calves, as well as for one of their common ancestors, the AI bull "E.". Consequently, samples were available for both parents in 11 cases, for the mother only in four cases, and neither parent in six cases. After identifying the candidate causal variant in the *GNPAT* gene and performing large-scale genotyping using a SNP array (see below), blood samples from three heterozygous carrier cows and three wild-type controls were collected in PAXgene Blood RNA Tubes (Qiagen) for RNA extraction and RT-PCR analysis. Finally, whole genome sequences, SNP array genotypes, and phenotypes from thousands to hundreds of thousands of animals across various breeds were also utilised in this study. Further details of this additional material and the analyses performed are provided below.

### Pedigree analysis

Detailed pedigree information was obtained from the French national pedigree database for 17 affected calves and 110,247 Aubrac controls, all of which were born between 2019 and 2021 and had both parents recorded. A search for common ancestry among the parents of the affected calves was performed using the anc_comm option of the pedig package [22]. Meanwhile, the genetic contribution of each ancestor to both the case and control populations was estimated using the prob_orig option of the same package. For individuals with a genetic contribution of 1% or greater in each population, the ratio of "contribution to the case population” to “contribution to the control population" was calculated.

### Necropsy and pathological examination

The frozen bodies of one control and three affected calves were subjected to digital radiography (XDR1, Canon Medical Systems), computed tomography (CT) scanning (80-slice CT scanner, Aquilion lighting, Canon Medical Systems), and necropsy at the National Veterinary School of Alfort. In addition to the classic post-mortem examination, special attention was given to the deformities of the skull and limb bones. The heads were sawed through the midline, and the left limbs were harvested, boiled, cleaned of residual soft tissue, and bleached with 5% hydrogen peroxide before partial skeletal reconstruction.

### DNA extraction

Genomic DNA was extracted from EDTA-treated blood using the Wizard Genomic DNA Purification Kit (Promega). DNA was also obtained from ear biopsies (collected post-mortem) and cryopreserved AI semen straws using the Gentra Puregene Cell and Tissue Kit (Qiagen). The purity and concentration of the DNA were assessed using a NanoDrop spectrophotometer (ThermoFisher Scientific).

### Homozygosity mapping

A total of 21 Aubrac cases, along with 26 of their parents and 1548 control animals from the same breed, were genotyped using various Illumina SNP arrays over time (Bovine SNP50, EuroG10K and EuroGMD; [23, 24]). The genotypes underwent parentage verification, phasing, and imputation to the Bovine SNP50 using FImpute3 [25] as part of the French genomic evaluation workflow, as detailed in the study by Mesbah-Uddin et al*.* [26]. The positions of the markers were based on the ARS-UCD1.2 bovine genome assembly.

We analysed sliding haplotypes composed of 20 markers (~ 1 Mb; incremented by one marker) for 44,574 informative markers (MAF > 0.01). Fisher’s exact tests were performed on 2 × 2 contingency tables that compared the number of animals homozygous for a given haplotype within a given window and the number of animals with any other genotype within that window in the case and control groups.

A Bonferroni correction was applied to account for multiple testing, given that there were n = 78,861 tests with at least one homozygous carrier in the case group. Consequently, a nominal type I error rate of α = 0.05 corresponded to a Bonferroni-corrected threshold of − log_10_(p) = 6.20.

Finally, adjacent overlapping haplotypes for which all cases were homozygous and none of the controls were homozygous were considered to establish the mapping interval. A haplotype test (of 35 contiguous markers in the present case) was used to predict the status at the RCDP locus (homozygous, heterozygous or wild type) for each animal with phased and imputed Illumina Bovine SNP50 genotyping data available.

### Analysis of whole genome sequences

The genomes of two calves affected by RCDP were sequenced with coverage rates of 19.2 × and 19.4 × using the Illumina NovaSeq6000 platform in 150 bp paired-end mode. Library preparation was performed using the NEXTflex PCR-Free DNA Sequencing Kit (Perkin Elmer Applied Genomics). The sequencing reads were aligned to the ARS-UCD1.2 bovine genome assembly [27] using the Burrows-Wheeler aligner (BWA-v0.6.1-r104; [28]) before identifying SNPs and small InDels with the GATK-HaplotypeCaller software [29], following the methods outlined in Daetwyler et al*.* and Boussaha et al*.* [13, 30].

Structural variations (SVs) were detected using the Pindel [31], Delly [32], and Lumpy [33] software. Putative SVs were recorded only if they were identified by at least two of these tools and if they exhibited a minimum of 70% mutual overlap within the same individual.

The variants were then compared to those found in 1867 control genomes from a previous study using the same methodology [6]. This control group included 39 Aubrac individuals, all non-carriers of the 35-marker haplotype common to affected calves, based on phased and imputed genotypes from the Illumina BovineSNP50 array. Additionally, the panel of control genomes comprised 1828 representatives from over 70 different cattle breeds or populations (see Additional file 1, Table S1), all considered homozygous wild-type during the filtering process, as recessive defects are mostly breed-specific, with rare exceptions (e.g. [34]).

In the search for the candidate causative variant, we focused on all SNPs, InDels, and SVs that met these criteria: (i) located within the interval identified by homozygosity mapping (Chr28:3,555,723–5,143,700 bp; see Results), (ii) observed in the homozygous state in both affected calves, and (iii) not segregating at all in the complete panel of control genomes.

The only remaining variant after filtering (in this case NC_037355.1:g.4039268G > A, see Results), was annotated using the Variant Effect Predictor (Ensembl release 110; https://www.ensembl.org/info/docs/tools/vep/index.html) [35], and visualised using the Integrative Genomics Viewer (IGV) [36].

### Validation of variant NC_037355.1:g.4039268G > A by Sanger sequencing and large-scale genotyping

As a first verification step, we genotyped the variant NC_037355.1:g.4039268G > A using PCR and Sanger sequencing in six Aubrac cattle: two affected calves, two obligate carriers (the parents of an affected calf), and two wild-type animals based on haplotype information.

We amplified a 640-bp segment encompassing the candidate variant using the primers 5′-TCCCTTCCTTCAAGGCTACA-3′ and 5′-GTTAGGAGCCAGAGCAGCAC-3′, and the Go-Taq Flexi DNA Polymerase (Promega), according to the manufacturer’s instructions. The PCR was performed in a Mastercycler pro thermocycler (Eppendorf). The program included an initial denaturation step at 95 °C for 5 min, followed by 35 cycles of denaturation at 95 °C for 30 s, annealing at 60 °C for 30 s, extension at 72 °C for 45 s, and a final extension step at 72 °C for 5 min.

The amplicons were purified and bidirectionally sequenced by Eurofins MWG (Hilden, Germany) using conventional Sanger sequencing. The resulting electropherograms were analysed with NovoSNP software for variant detection [37].

In a second step, to genotype this variant on a larger scale, we added a probe to the Illumina EuroGMD SNP array, designed as follows: TTTGTTCAGTAGGAAGTGAGGGCAGCCATTTTGAGCATAACATGATTCTCAGTGTTTTTC[A/G]NNCTTGCCGCATGCACTTTTGTTTAAATGTGAGGAGAGTATGGCTGTATACAAAGTGAAA. At the time of writing, EuroGMD SNP array genotypes were available from 21 affected calves analysed for this study and 376,730 unaffected animals from 19 French breeds (including 1195 Aubrac cattle) genotyped as part of routine genomic evaluations.

### Minigenes construction

The canonical transcript of the bovine *GNPAT* gene (ENSBTAT00000066521) was utilised to determine a 1149 bp fragment containing exon 11, intron 11 and exon 12 of the gene. This fragment was amplified from the genomic DNA of a calf homozygous for the NC_037355.1:g.4039268A allele.

BamHI and XhoI restriction sites were incorporated into primers 5′-TACCGAGCTCGGATCCTCCAGAGGATGTCTACAGTTGC-3′ and 5′-GCCCTCTAGACTCGAGTTGCAAAGATTTACACACCTGA-3′ designed for this purpose.

The PCR was performed in a 25 μL reaction mixture that contained 12.5 µL of 2X KAPA HiFi HotStart ReadyMix (Roche), 50 ng genomic DNA, and 0.3 μM of each primer. The PCR program included an initial denaturation at 95 °C for 3 min, followed by 30 cycles of denaturation at 98 °C for 20 s, annealing at 65 °C for 15 s, extension at 72 °C for 1 min, and a final extension at 72 °C for 1 min 20 s.

The PCR products were then cloned into the pcDNA3.1(+) vector (Invitrogen), linearised by the BamHI and XhoI restriction enzymes, using the T4 DNA Ligase (New England Biolabs), following the manufacturer’s instructions. The resulting minigene construct carrying the alternative g.4039268A allele was designated as pcDNA3.1-GNPAT_A.

Subsequently, the g.4039268G reference allele was introduced into the pcDNA3.1-GNPAT_A minigene construct through site-directed mutagenesis, resulting in the pcDNA3.1-GNPAT_G minigene construct. This process utilised the QuikChange II XL Site-Directed Mutagenesis Kit (Agilent) using primers 5′-CTCAGTGTTTTTCGGACTTGCCGCATGC-3′ and 5′-GCATGCGGCAAGTCCGAAAAACACTGAG-3′ following the manufacturer’s instructions.

The sequences of both minigenes were verified by Sanger sequencing using T7 and BGH universal primers and the additional primer 5′-CAAGTGGGTCTGGGGTCTG-3′.

### Cell culture and transfection

Human embryonic kidney (HEK) 293T cells were maintained in DMEM supplemented with 10% fetal calf serum (Gibco). The cells were seeded at a density of 300,000 cells per well in 6-well plates. Twenty-four hours later, they were transfected with 1 µg of each minigene construct mixed with 3 µL of Lipofectamine 2000 Reagent (Invitrogen) per well, according to the manufacturer’s instructions. Four hours after transfection, the medium was replaced with DMEM supplemented with 10% fetal bovine serum and the cells were incubated at 37 °C with 5% CO2. Forty-eight hours post-transfection, the cells were washed with phosphate-buffered saline (PBS) and lysed using RLT buffer (Qiagen).

### RNA extraction from cell culture and RT-PCR analysis

Total RNA was extracted from lysed transfected cells using the RNeasy Mini Kit according to the manufacturer’s instructions (Qiagen). The reverse transcription (RT) step was conducted using the SuperScript III First-Strand Synthesis System for RT-PCR (Invitrogen) with 400 ng RNA, OligodT20 5 µM, 500 µM of each dNTP, MgCl_2_ 5 mM, DTT 0.01 M, 40 U RNaseOUT and 200 U SuperScript III following the manufacturer’s instruction. The RT products were treated with 2 U RNase H for 20 min at 37 °C.

The PCR step was achieved using primer T7 (5′-TAATACGACTCACTATAGGG-3′) and BGH (5′-TAGAAGGCACAGTCGAGG-3′) located within the 5′- and 3′-untranslated regions of pcDNA3.1-GNPAT minigene constructs, respectively. The reaction was performed in a 50 μL mixture containing 1.25 U GoTaq DNA polymerase (Promega), 200 μM of each dNTP, 2 μL cDNA and 0.5 μM of each primer. The PCR program consisted of an initial denaturation step at 95 °C for 2 min, followed by 30 cycles of denaturation at 95 °C for 30 s, annealing at 55 °C for 30 s, extension at 72 °C for 1 min 30 s, and a final extension step at 72 °C for 5 min.

### RNA extraction from blood and RT-PCR analysis

RNA was extracted from blood samples collected in PAXgene Blood RNA Tubes using the PAXgene Blood RNA Kit (Qiagen). The tubes were stored at -20 °C for 1 month and then thawed at room temperature for 2 h before RNA extraction. The samples were centrifuged for 10 min at 4000*g*, and the supernatant was carefully discarded. RNA was extracted from cell pellets following the manufacturer’s guidelines, and the purity and integrity of the RNA was assessed using the Bioanalyzer 2100 (Agilent). A commercial sample of bovine muscle RNA (Gentaur), amounting to 1 µg, was used as a positive control.

The (RT) step was conducted using the SuperScript III First-Strand Synthesis System for RT-PCR with 60 ng RNA, OligodT20 5 µM, 500 µM each dNTP, MgCl_2_ 5 mM, DTT 0.01 M, 40 U RNaseOUT and 200 U SuperScript III following the manufacturer’s instruction. The RT products were treated with 2 U RNase H for 20 min at 37 °C.

The PCR step was achieved using primers 5′-GCTTTCGCTTCCTATGCAGT-3′ and 5’-TGTCCCTCGTCATCACTTGT-3′ located within the *GNPAT* exons 11 and 12. Each cDNA sample was amplified in quadruplicate in a 25 μL mixture containing 0.75 U GoTaq DNA polymerase (Promega), 200 μM of each dNTP, 1 μL cDNA and 0.5 μM of each primer. The PCR program started with an initial denaturation at 95 °C for 2 min, followed by 45 cycles of denaturation at 95 °C for 30 s, annealing at 53 °C for 30 s, extension at 72 °C for 1 min, and a final extension step at 72 °C for 10 min. The four PCR replicates obtained from each sample were pooled and concentrated with the MinElute PCR Purification Kit (Qiagen) before proceeding to gel electrophoresis.

### Prediction of exonic splicing enhancers (ESE) and protein structure analysis

ESE motif prediction was conducted using ESEfinder 3.0 software in the context of the A and G alleles. This allowed us to identify the creation or disruption of putative splicing regulatory elements (http://krainer01.cshl.edu/cgi-bin/tools/ESE3/esefinder.cgi?process=home; [38, 39]). The coding sequences of normal and abnormal *GNPAT* transcripts were translated into amino acid sequences using ExpASy (https://web.expasy.org/translate/; [40]). Information regarding protein domains was obtained from UniProt (https://www.uniprot.org/uniprotkb/A4IF87/entry, accessed 08.05.2020) and from the work of Ofman et al*.* [18].

### Effects of the NC_037355.1:g.4039268A allele on juvenile mortality rates

The phenotypic effects of four mating types on juvenile mortality rates were evaluated using records from the French National Bovine Database and the following fixed-effect model:

$${\text{y}}_{\text{ij}}=\upmu +{\text{m}}_{\text{j }}+ {\text{e}}_{\text{ij}}$$

where y_ij_ represents the phenotype of interest, μ is the overall phenotypic mean, m_j_ is the fixed effect of the mating type, and e_ij_ is the random residual error. The analysis used the GLM procedure in SAS software (version 9.4; SAS Institute Inc., Cary, NC).

The mating types examined were named 1 × 1, 1 × 0, 0 × 1, and 0 × 0, where the first position indicates the genotype of the sire and the second position indicates the genotype of the maternal grandsire in terms of allele dosage for the g.4039268A allele. These genotypes were determined through direct genotyping of the variant using the Illumina EuroGMD SNP array for 10% of the sires and 3% of the maternal grandsires. For the remaining individuals, genotypes were inferred based on the 35-marker merged haplotype (from positions Chr28:3,583,342 bp to Chr28:5,092,017 bp on the ARS-UCD1.2 bovine reference genome assembly [27]) identified through homozygosity mapping (see Results).

Juvenile mortality was examined during four periods commonly referenced in the literature (0–2, 3–14, 15–55 and 56–365 days after birth; e.g. [41, 42]). Additionally, we analysed a combination of the first two periods. For each time window, the mortality rate was calculated by dividing the number of calves that died of natural causes during the period by the number of calves present at the beginning of that period.

We also assessed the expected impact on juvenile mortality rates, assuming complete penetrance of lethality under homozygosity. This was done using the formula $\frac{1}{4 \left(2-fA\right)}(1-\mu )$ adapted from Michot et al*.* [43], where fA is the population frequency of the g.4039268A allele, and μ is the phenotypic mean of the trait (i.e., the mortality rate for a given period).

The following reasoning obtains this formula. The theoretical possible genotypes for the ungenotyped dams in at-risk mating (which are daughters of heterozygous bulls) can be classified as wild-type (with a probability of $\frac{1}{2}(1-fA)$), heterozygous ($\frac{1}{2}$) and homozygous ($\frac{1}{2}fA$). However, if we assume the complete penetrance of perinatal lethality under homozygosity, homozygous individuals would not survive past birth or shortly thereafter. Thus, the possible genotypes for these dams include only wild-type and heterozygous, with respective probabilities of $\frac{1-fA}{2 (1-\frac{1}{2}fA)}$ and $\frac{1}{2 (1-\frac{1}{2}fA)}$ (i.e. their initial probability divided by 1 minus the probability of the homozygous genotype).

Thus, the probability that mating a heterozygous sire with the ungenotyped daughter of another heterozygous bull will result in the birth of a homozygous calf is $\frac{1}{4 (2-fA)}$ (i.e., the probability that the dam is heterozygous ($\frac{1}{2 (1-\frac{1}{2}fA)}$), multiplied by the probability that it will transmit the deleterious variant ($\frac{1}{2}$) and multiplied by the probability that the heterozygous sire to which it is bred will also transmit the deleterious allele ($\frac{1}{2}$)). Finally, the mortality is increased by $\frac{1}{4 (2-fA)}$ multiplied by (1-µ).

### Effects of heterozygosity for the NC_037355.1:g.4039268A allele on performance traits

The Aubrac is one of the nine breeds included in the French national genetic evaluation of beef cattle (see https://www.geneval.fr/_files/ugd/a0004f_9157941e10384a35bf98c06818155506.pdf for details). Each year, animals are assessed using a national polygenic BLUP evaluation for five traits measured on the farm: birth weight, ease of calving, muscular development, skeletal development, and weight at 210 days.

Phenotypes of genotyped animals were extracted from the national database and adjusted for non-genetic effects as estimated in the French national genetic evaluation. The effect of the NC_037355.1:g.4039268A allele was tested using the GWAS method for the five traits being studied, utilising GCTA software version 1.26 [44]. The analysis was performed with the mlma option and applied to the following mixed linear model:

$$\mathbf{y}=\boldsymbol{1}\upmu +\mathbf{x}\text{b}+\mathbf{u}+ \mathbf{e}$$

where $\mathbf{y}$ is the vector of corrected phenotypes; $\upmu$ is the overall mean; $\boldsymbol{1}$ is a vector of ones; b is the additive effect of the derived allele; **x** is the genotype for the SNP; $\mathbf{u}\sim N(\boldsymbol{0},\mathbf{G} {\upsigma }_{\text{u}}^{2})$ is the vector of random polygenic effect, where $\mathbf{G}$ is the genomic relationship matrix calculated using the 50K SNP genotypes (computed without SNPs from chromosome 28), and ${\upsigma }_{\text{u}}^{2}$ is the polygenic variance that is estimated based on the null model without the SNP effect; and $\mathbf{e}\sim N(\boldsymbol{0},\mathbf{I} {\upsigma }_{\text{e}}^{2})$ is the vector of random residual effects, where $\mathbf{I}$ is the identity matrix and ${\upsigma }_{\text{e}}^{2}$ is the residual variance.

Depending on the age at recording and assessment method (visual scoring or weighing), the number of animals analysed per trait ranged from 4401 to 8416. Of these, 44% had their status determined by direct genotyping of the variant and 56% by the 35-marker haplotype test mentioned above.

### False discovery rate control

To account for multiple testing, we applied the Benjamini–Hochberg procedure to the raw p-values obtained from analysing the effects of the NC_037355.1:g.4039268A allele on various performance traits and on juvenile mortality rates across different time periods. The formula used is $q=p\frac{n}{k}$, where q is the Benjamini–Hochberg q-value, p is the raw p-value, n is the total number of hypotheses tested, and k is the rank of the raw p-value in ascending order. We set the threshold at q = 0.05.

## Results

### Pedigree analysis suggests an autosomal recessive mode of inheritance

The study examined eight male and thirteen female chondrodysplastic calves, all born to unaffected parents over an 18-year period, in 21 purebred Aubrac herds across France. An analysis of the pedigrees for 17 of these cases, with parental information dating back to the 1960s, revealed several recent inbreeding loops indicative of an autosomal recessive mode of inheritance. However, it did not allow for the identification of a single common ancestor among all their parents.

**Fig. 1:**
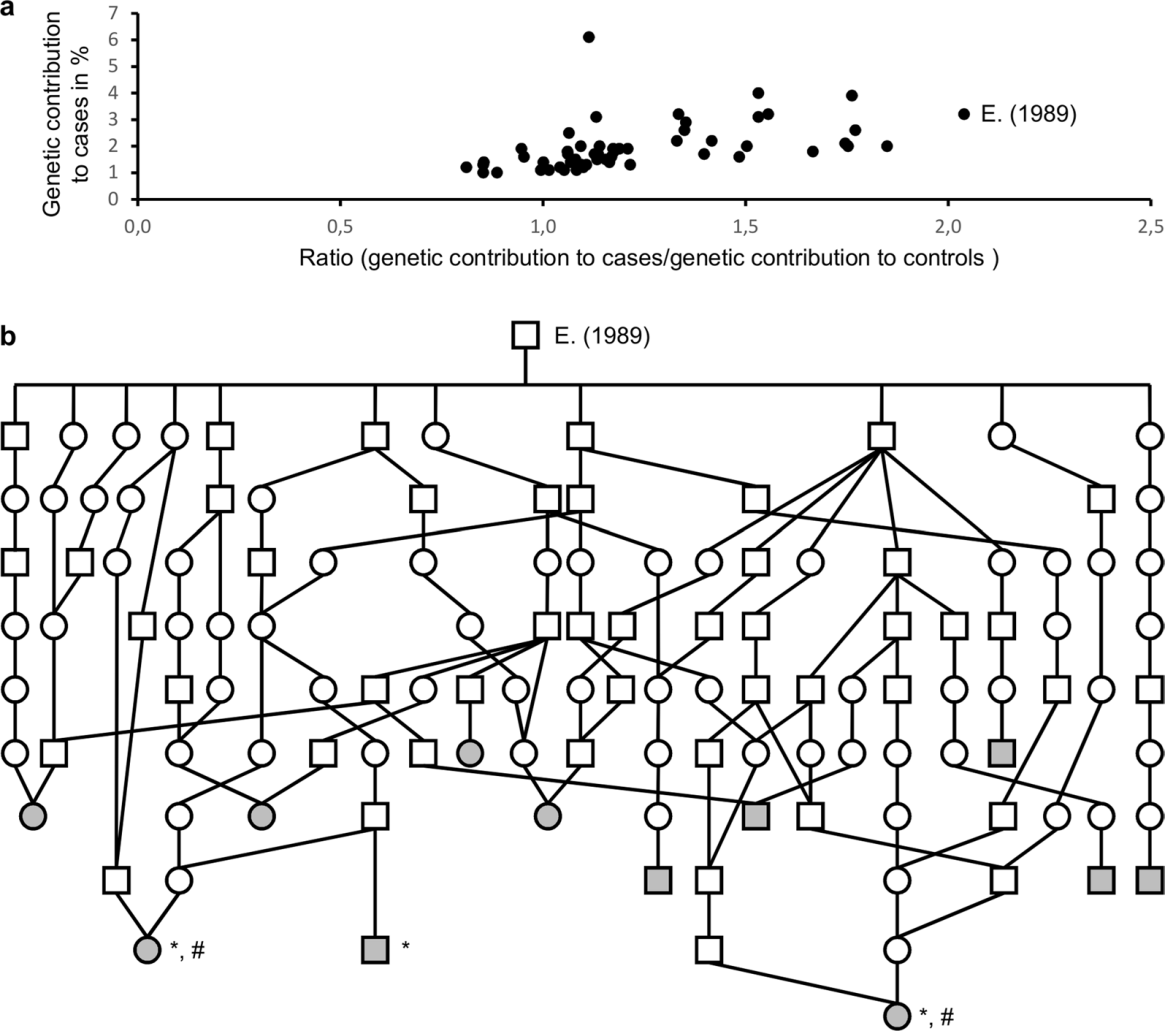
Pedigree analysis. **a** Graph showing the genetic contribution to the case group (n = 17) and the ratio "contribution to the cases/contribution to 110,247 controls" for ancestors with a genetic contribution greater than or equal to 1% in each population. **b** Simplified pedigree of 12 cases descending from ancestor “E.”. Squares and circles represent males and females, respectively. Gray-filled symbols correspond to affected calves. *: Animals necropsied. #: Individuals selected for whole genome sequencing

Further analysis highlighted the bull “E.” (born in 1989) as the most important source of this putative recessive defect in recent decades. Notably, “E.” exhibited a ratio of 2.04 between its genetic contributions to the case group (3.20%) and a control group of 110,247 individuals born between 2019 and 2021 (1.57%; Fig. 1a). In contrast, the mean and standard deviation for this ratio among 54 ancestors with a genetic contribution of 1% or more in each population were 1.23 and 0.28, respectively. Additionally, this AI bull “E.” appeared in the pedigrees of 12 out of the 17 cases (Fig. 1b).

### Clinical findings are compatible with RCDP

**Fig. 2:**
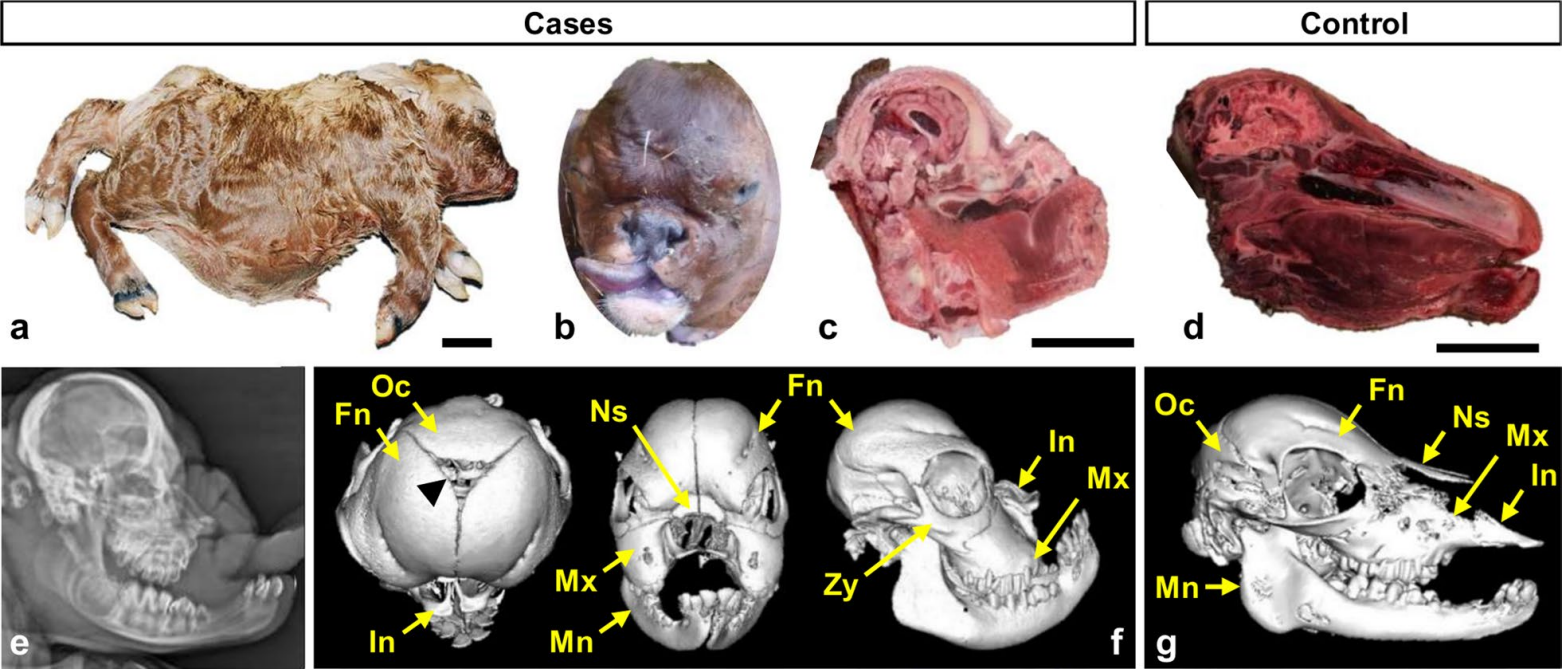
Macroscopic view of Aubrac chondrodysplastic calves and characterisation of their craniofacial dysmorphism. **a**, **b** General view and detail of the head of affected calves with facial features reminiscent of the French Bulldog dog breed, hence the name “bulldog calf” initially given to this condition by breeders. **c**,** d** Longitudinal section of the skull of a case and a control calf, respectively. **e** Radiograph of the head of an affected calf. **f**, **g** CT scan images of the head of a case and a control calf, respectively. The black arrowhead points to the anterior fontanelle between the occipital bone and the two frontal bones observed in affected calves. Fn: Frontal bone. In: Incisive bone. Mn: Mandible. Mx: Maxillary bone. Ns: Nasal bone. Oc: Occipital bone. Zy: Zygomatic bone. Scale bars = 10 cm

The 21 affected calves were stillborn and displayed extremely disproportionate dwarfism characterised by craniofacial dysmorphism, short limbs with hypermobile joints, a distended abdomen prone to eventration, and low birth weights despite standard gestation length (i.e., ~ 20–30 kg versus ~ 40 kg; Fig. 2). Only three of the affected calves (two females, one male) were available for extensive pathological examination.

Radiographs, CT scans, and longitudinal skull sections were used to characterise the craniofacial dysmorphism better. This condition primarily involved severe hypoplasia of the maxilla, leading to secondary deformities in neighbouring bones and soft tissue structures. These deformities included cleft palate, curvature of the mandible, protrusion of the tongue, bossing of the frontal bone, and the presence of an anterior fontanelle (Fig. 2).

**Fig. 3:**
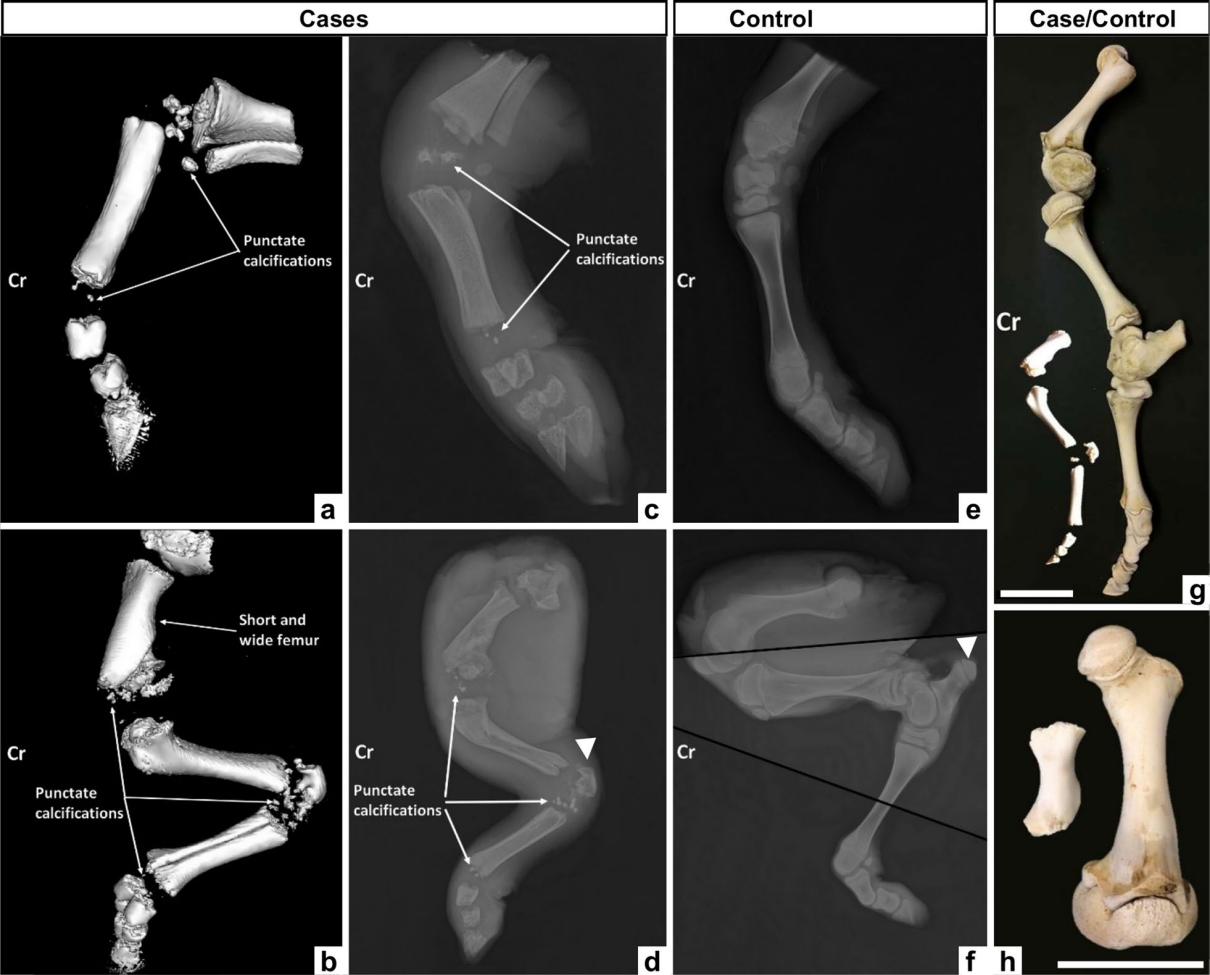
Imaging and skeletal preparation of the limbs of chondrodysplastic and control calves. **a**, **b** CT scans of the anterior (**a**) and posterior (**b**) limbs of the case. **c**, **d** Radiographs of the limbs of the same RCDP-affected individual. **e**, **f** Radiographs of a control individual's anterior (**e**) and posterior (**f**) limbs. Note that panel (**f**) is a composite of 3 radiographs of the same posterior limb (with fusion lines in black), as its length and frozen state in an extended position did not allow the same position as in the matched case limb to be reproduced in a single image. **g** Skeletal preparation of the left hind limb of case and control calves. **h** Detail of the humerus shown in **g**. Note the presence of multiple punctate calcifications where the epiphyses should be found and the absence of the femoral head (visible in **h**) and the tuberosity of the calcaneus (indicated by a white arrowhead in **d** and **f**). Cr: Cranial orientation. Scale bars = 10 cm

Imaging and skeletal preparation also revealed platyspondyly of the thoracic and lumbar vertebrae, abnormally short ribs, and rhizomelic limb shortening (see Additional file 2, Figure S1) (Fig. 3). Specifically, the proximal long bones had shortened diaphyses and enlarged metaphyses with thickened cortices. In contrast, the diaphyses of the distal long bones (metatarsus, metacarpus, and phalanges) were properly developed. Additionally, the tuberosity of the calcaneus, the femoral head, and all epiphyses were either absent or reduced to punctate calcifications (Fig. 3).

Finally, the necropsy revealed hyperlaxity of all joints except the stifle and hock, which were affected by arthrogryposis. There were no notable malformations of the internal organs. Based on all these elements, we diagnosed the calves with rhizomelic chondrodysplasia punctata (RCDP).

### Mapping and identification of a candidate causal variant in *GNPAT*

**Fig. 4:**
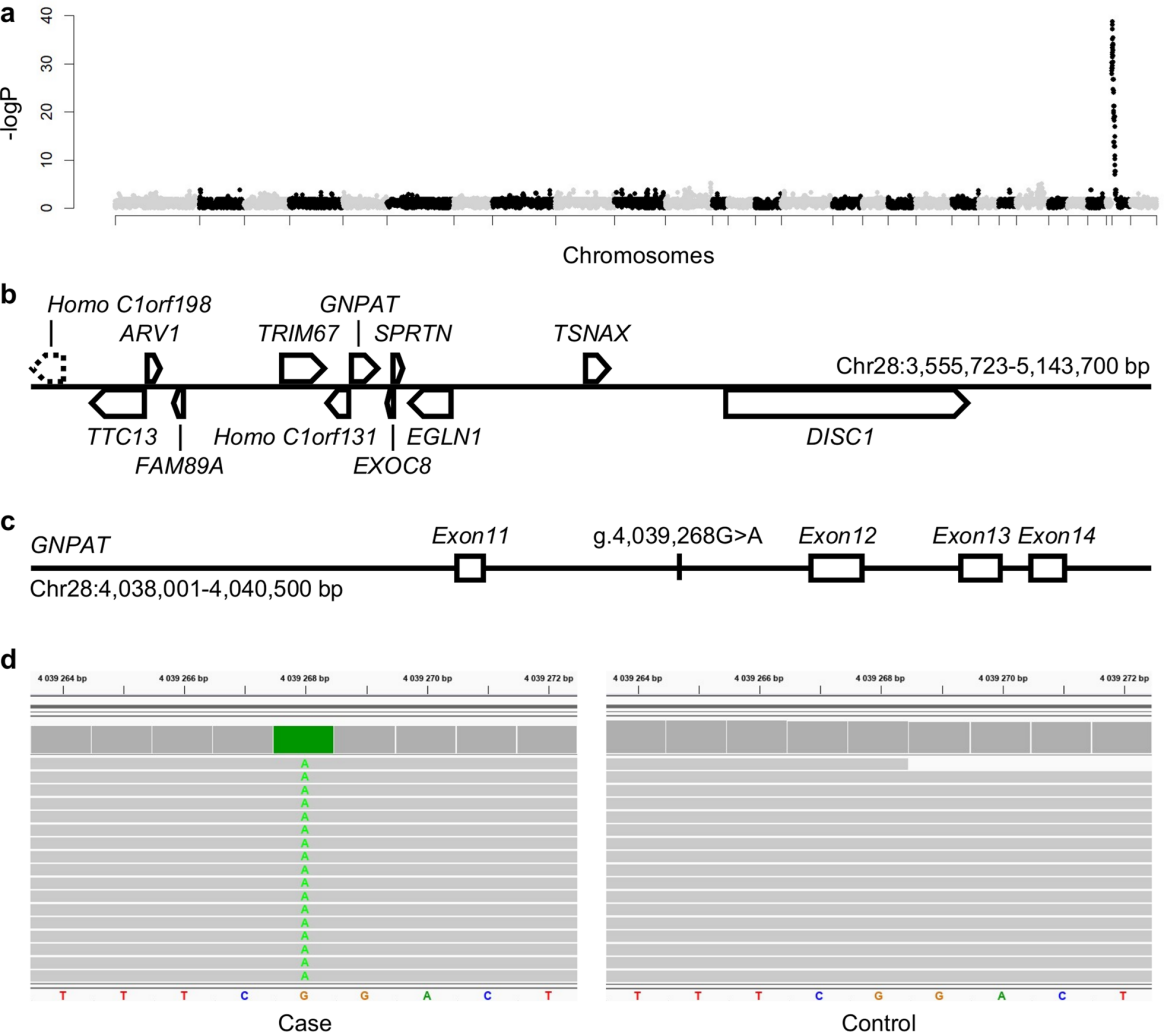
Mapping and identification of a candidate causative variant in *GNPAT* intron 11. **a** Manhattan plot of homozygosity mapping results with phased and imputed Illumina BovineSNP50 array genotypes from 21 RDCP-affected and 1548 control individuals. The 0.05 significance threshold was set at –log_10_(P) = 6.20 after Bonferroni correction of Fisher's exact test p-values. **b** Details of the genes located within the mapping interval. The dashed line indicates that the Homo C1orf198 gene encompasses the left border of the interval. **c** Details of the localisation of the NC_037355.1:g.4039268G > A candidate variant in the 11th intron of *GNPAT*. **d** Integrative Genomics Viewer screenshot showing homozygosity for this substitution in the whole genome sequence of an affected calf compared to a control

To gain insight into the molecular causes of this bovine form of RCDP, we first used a homozygosity mapping approach. We analysed the Illumina BovineSNP50 genotypes of 21 affected animals alongside 1628 controls, using sliding windows of 20 markers and mapped the RCDP locus to the beginning of chromosome 28 (Fig. 4a).

Under the peak position, we identified a 35-marker haplotype between positions Chr28:3,583,342 bp and Chr28:5,092,017 bp on the bovine reference genome assembly ARS-UCD1.2. This haplotype was found in the homozygous state in all affected animals and none of the controls. The most proximal markers outside of this segment defined the boundaries of a 1.6 Mb mapping interval (Chr28:3,555,723–5,143,700 bp), which contains *GNPAT* and 11 additional genes: *Homo C1orf198, TTC13, ARV1, FAM89A, TRIM67, Homo C1orf131, EXOC8, SPRTN, EGLN1, TNSAX,* and *DISC1* (Fig. 4b).

We then sequenced the complete genomes of two RCDP-affected calves. We identified a total of 3115 sequence variants within the mapping interval, for which both calves were homozygous for the alternative allele (see Additional file 3, Table S2). As an initial step, we filtered out the variants found in 39 non-carrier Aubrac individuals based on haplotype information, leaving us with just seven candidate variants. Considering that, with rare exceptions, most recessive defects result from recent inbreeding and are specific to a single breed, we eliminated variants observed in 1827 genomes from more than 70 different breeds. This process narrowed our list down to a single candidate: a deep intronic substitution located 549 bp downstream and 323 bp upstream of *GNPAT* exons 11 and 12, respectively (NC_037355.1:g.4039268G > A; Fig. 4 c, d). It should be noted that deep intronic variants are, by definition, located within an intron and more than 100 bp away from the nearest exon–intron boundary.

### Validation of the *GNPAT* NC_037355.1:g.4039268G > A variant through large-scale genotyping

As a first verification, we genotyped the *GNPAT* NC_037355.1:g.4039268G > A variant in the 21 cases, all their available parents (n = 26) and the AI bull “E.” using the Illumina EuroGMD custom SNP array. As anticipated, all affected calves were homozygous for the derived allele, while all unaffected parents and bull “E.” were heterozygous.

For further validation, we expanded our analysis to 1195 unaffected Aubrac cattle and 375,535 controls from 17 different breeds, all of which were genotyped on the same array for genomic evaluation purposes. The NC_037355.1:g.4039268A allele was present exclusively in Aubrac cattle, where it was found to have a frequency of 2.6%. None of these Aubrac animals was homozygous for the derived allele (Table 1).

**Table 1:** Results of large-scale genotyping of the *GNPAT* NC_037355.1:g.4039268G > A variant in 376,730 unaffected animals from 18 breeds

Breeds	Genotypes			
	GG	AG	AA	f(A)
Abondance	5789	–	–	0
Aubrac	1136	59^a^	–	2.60
Blonde d'Aquitaine	6114	–	–	0
Bretonne pie noir	43	–	–	0
Brown swiss	4648	–	–	0
Charolaise	11,896	–	–	0
Créole	91	–	–	0
Holstein	243,189	–	–	0
Jersey	2991	–	–	0
Limousine	2391	–	–	0
Montbéliarde	141,296	–	–	0
Normande	31,745	–	–	0
Parthenaise	1005	–	–	0
Rouge des prés	17	–	–	0
Salers	1007	–	–	0
Simmental	4914	–	–	0
Tarentaise	2994	–	–	0
Vosgienne	758	–	–	0

The number of animals is given for each genotype per breed; f(A): frequency of the NC_037355.1:g.4039268A allele.^a^ These 59 animals do not include any of the obligate carriers (parents of affected animals)

### The NC_037355.1:g.4039268A allele activates cryptic splice sites in *GNPAT* intron 11

Following these preliminary verifications, we conducted a series of analyses to explore the effects of the NC_037355.1:g.4039268A allele on *GNPAT* function. Because tissues from RCDP-affected calves were collected and frozen at − 20 °C before the discovery of the candidate variant, they were not available for subsequent RNA extraction. As a result, we attempted to perform a Western blot analysis using an antibody targeting the N-terminal region of the GNPAT protein (ab75060, Abcam). However, we could not detect a signal at the expected molecular weight in wild-type samples, whether using proteins extracted from the muscle of a control animal in our laboratory or a commercial extract (BT-102, GENTAUR; results not shown). Although this antibody has proven effective against both human and mouse GNPAT [45, 46], we concluded that it does not work with the bovine orthologous protein.

**Fig. 5:**
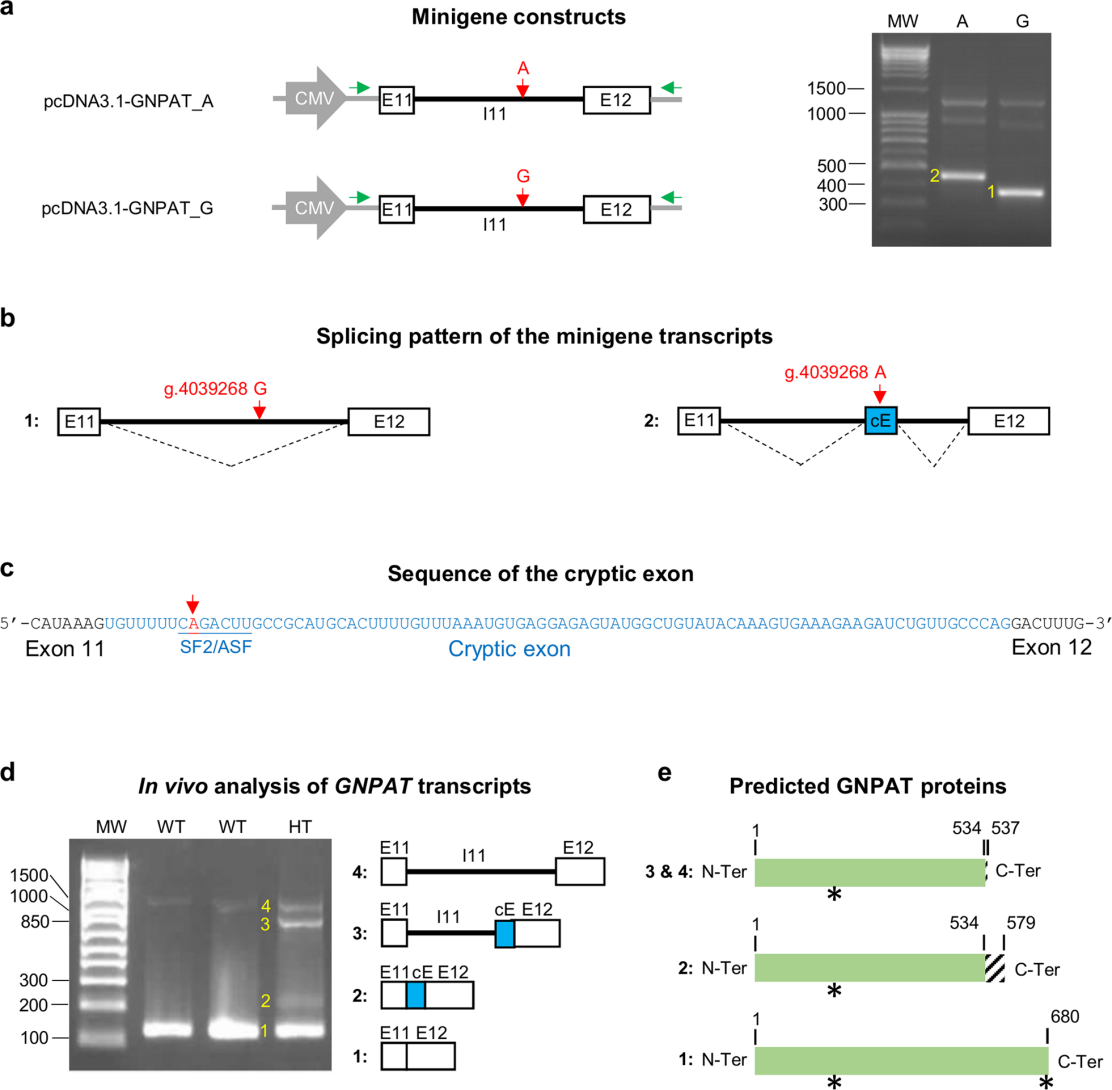
In vitro, in silico and in vivo analysis of the effect of the NC_037355.1:g.4039268G > A substitution on the *GNPAT* splicing. **a** Minigene analysis: Illustration of the pcDNA3.1-GNPAT minigenes carrying the derived or ancestral alleles in *GNPAT* intron 11 (upper and lower left panels, respectively) and results of the RT-PCR and gel electrophoresis after transfection of HEK293T cells with the pcDNA3.1-GNPAT_A (A) and pcDNA3.1-GNPAT_G (G) minigenes (right panel). cDNAs were amplified with primers targeting the pcDNA3.1 5'UTR and 3'UTR regions (green arrows). Two major PCR products were detected (labelled 1 and 2). MW: Molecular weight. (**b**) Splicing patterns associated with the two minigenes based on Sanger sequencing of the amplicons shown in **a**. cE: Cryptic exon. (**c**) Sequence details of the cryptic exon: The NC_037355.1:g.4039268G > A substitution increases the score for a predicted SF2/ASF binding site located in its 5' region according to the ESEfinder 3.0 software. **d** In vivo analysis of *GNPAT* transcripts. Left panel, representative subset of the results obtained after gel electrophoresis following RT-PCR on total blood RNA extracted from wild-type (WT) and heterozygous (HT) Aubrac cattle using primers targeting *GNPAT* exons 11 and 12. Four observed PCR products are numbered, and their structures are shown (see text for details). MW: Molecular weight. **e** Consequences of the splicing patterns shown in **d** on the primary structure of the GNPAT protein. Normal amino acids (AAs) are shown in green, and novel AAs are shaded. The acyltransferase motif (AAs 162 to 167) and the peroxisomal targeting signal 1 (PTS1, AAs 678 to 680; [18]) are marked with an asterisk

Following our initial unsuccessful attempt, we conducted a minigene analysis to explore the potential impact of the NC_037355.1:g.4039268A allele on *GNPAT* splicing in vitro. We constructed two expression plasmids containing exon 11, intron 11, and exon 12 of the *GNPAT* gene, one with the ancestral allele and the other with the derived allele from the deep intronic variant (pcDNA3.1-GNPAT_G and pcDNA3.1-GNPAT_A, respectively; Fig. 5a).

RT-PCR analysis of HEK293T cells transfected with both minigenes revealed only one major specific transcript for each construct, but of different sizes (Fig. 5a). Sanger sequencing of the amplicons indicated that transcript No. 1 from pcDNA3.1-GNPAT_G was fully spliced, corresponding to exons 11 and 12. In contrast, transcript No. 2 from pcDNA3.1-GNPAT_A corresponded to exon 11 and exon 12 separated by a small segment of intron 11, which contained an 86 bp cryptic exon (Chr28:4,039,260–4,039,345; Fig. 5b, c).

Consistent with these findings, sequence analysis using the ESEfinder 3.0 software revealed that the A allele was predicted to increase the binding capacity of the SF2/ASF splicing factor at the 5′ end of the cryptic exon, potentially explaining the selective inclusion of the latter in transcript No. 2 (Fig. 5c).

To further investigate the effects of the deep intronic variant in vivo, we performed RT-PCR analyses on total blood RNA extracted from three heterozygous and three wild-type Aubrac cattle. cDNA amplification using primers that target *GNPAT* exons 11 and 12, followed by agarose gel electrophoresis, produced four distinct bands (Fig. 5d). These were purified and sequenced using Sanger’s method.

Bands 1 and 4 were observed in both wild-type and heterozygous animals. They corresponded to amplicons with exons 11 and 12, consisting of either fully spliced or unspliced intron 11, respectively. Conversely, bands 2 and 3 were present exclusively in heterozygous animals and resulted from abnormal splicing of intron 11. Between exons 11 and 12, PCR product 2 contained the same 86 bp cryptic exon observed in the minigene analysis. In contrast, PCR product 3 included the portion of intron 11 located between exon 11 and the cryptic exon, in addition to the latter.

The combined results of our in vitro, in silico and in vivo analyses indicate that the NC_037355.1:g.4039268A allele affects *GNPAT* splicing by activating cryptic splice sites within intron 11. The incorporation of all or part of intron 11 into the *GNPAT* mRNA is predicted to cause frameshifts and generate mutant proteins lacking the last 21% amino acids of the bovine GNPAT protein, and in particular, the C-terminal microbody targeting signal (Fig. 5e).

### Mining the large dataset of records from the French National Bovine Database to study the effects of the NC_037355.1:g.4039268A allele

As a final step in our study, we took advantage of the large dataset of records from the French National Bovine Database to gain further insight into the phenotypic effects of the NC_037355.1:g.4039268A allele in both heterozygous and homozygous states. We first investigated the penetrance and expressivity of this allele by examining juvenile mortality rates associated with different mating combinations between genotyped sires and daughters of genotyped sires at four different ages, using a fixed effect model (Table 2).

**Table 2:** Analysis of five juvenile mortality rates for different mating types for the NC_037355.1:g.4039268G > A variant

Trait	Mating type	N	Raw mean (%)	Diff (%)	SE (%)	Raw p-value	BH q-value1	BH q-value2
56–365 days mortality rate	1 × 1	268	1.49	0.47	0.62	0.44	0.73	0.48
	1 × 0	4078	1.08	0.06	0.16	0.72	0.77	Na
	0 × 1	4973	0.95	− 0.08	0.15	0.60	0.73	Na
	0 × 0	91,703	1.02	–	–	–		-
15–55 days mortality rate	1 × 1	271	1.11	0.37	0.53	0.48	0.73	0.48
	1 × 0	4111	0.80	0.07	0.14	0.63	0.73	Na
	0 × 1	5022	0.94	0.20	0.13	0.11	0.32	Na
	0 × 0	92,397	0.74	–	–	–		-
3–14 days mortality rate	1 × 1	277	2.17	1.28	0.57	0.02	0.10	0.03
	1 × 0	4144	0.80	-0.09	0.15	0.54	0.73	Na
	0 × 1	5077	1.08	0.20	0.14	0.15	0.32	Na
	0 × 0	93,227	0.89	–	–	–		–
0–2 days mortality rate	1 × 1	300	7.67	5.69	0.81	1.59e^−11^	1.20e^−10^	3.98^e−11^
	1 × 0	4248	2.45	0.47	0.22	0.03	0.11	Na
	0 × 1	5178	1.95	− 0.03	0.20	0.89	0.89	Na
	0 × 0	95,109	1.98	–	–	–		-
0–14 days mortality rate	1 × 1	300	9.67	6.82	0.97	1.32e^−11^	1.20e^−10^	3.98^e−11^
	1 × 0	4248	3.23	0.38	0.26	0.15	0.32	Na
	0 × 1	5178	3.01	0.16	0.24	0.49	0.73	Na
	0 × 0	95,109	2.85	–	–	–		–

“Mating” indicates the genotype for the NC_037355.1:g.4039268G > A variant in allelic dosage (1 = heterozygous carrier of the mutant allele; 0 = non-carrier) of the sire and maternal grandsire of the group of individuals considered. For example, “1 × 0” refers to the progeny of a carrier bull with the daughter of a non-carrier bull. N: Number of observations. SE: Standard error. Diff (%): Difference between the mating type studied and the control group (i.e., mating type 0 × 0). Raw p-value: Student's t-test. Note the significant differences between the 1 × 1 and 0 × 0 genotype groups for several periods. Note also that we found a slight but suggestive + 0.47 increase in mortality for the 0–2 day period in matings between carrier sires and non-carrier sires (1 × 0 vs 0 × 0) because the mutant allele segregates at a frequency of 2.60% in the maternal grand-dam population (raw p-value = 0.03). BH q-value1: Benjamini–Hochberg q-value taking into account all tests performed. BH q-value2: Benjamini–Hochberg q-value considering only the comparisons between mating types 1 × 1 and 0 × 0, assuming that the candidate causal variant has a recessive effect

As anticipated, we observed a significant increase in mortality rates for at-risk matings (where both the sire and maternal grandsire are heterozygous; 1 × 1) compared to control matings (with a wild-type sire and maternal grandsire; 0 × 0) during the 0–2 day period (+ 5.69%; Benjamini–Hochberg q-value < 1.20e−10). Additionally, depending on the multiple testing correction applied (see Table 2 legend and Discussion), we noted a significant or suggestive effect for the 3–14 day period (+ 1.28%; raw p-value = 0.02 and Benjamini–Hochberg q-values of 0.03 or 0.10). This suggests that some homozygous mutant calves were not stillborn but died a few days after birth.

Overall, combining these periods, the increase in mortality reached + 6.82% within the first two weeks after birth (Benjamini–Hochberg q-value < 1.20e−10). This figure amounts to about half of the expected + 12.30% increase in mortality in at-risk matings, assuming complete penetrance (see Methods for calculation details).

To determine whether this discrepancy between the expected and observed increase in the mortality rate was due to incomplete penetrance or to underreporting of stillbirths, we reviewed the 21 cases reported to the ONAB. Only 61.90% (13/21) had been ear-tagged and officially recorded in the French national bovine database. When we compared the proportions “observed/expected increase in mortality within the first two weeks” (+ 6.82%/ + 12.30% = 55.44%) and “ear-tagged individuals/reported cases” (61.90%), using a χ-squared goodness-of-fit test, the results (12:9 vs 13:8; p = 1) indicated that these two proportions were not significantly different. Thus, we concluded that the penetrance of peri- and postnatal mortality is likely complete in homozygous carriers of the NC_037355.1:g.4039268A allele.

Finally, we investigated the effects of the NC_037355.1:g.4039268A allele on five performance traits evaluated each year within the national polygenic BLUP evaluation framework. Our findings indicated a suggestive result for only one trait: a reduction of one point in muscular development (MDev) at the age of 210 days in heterozygous carriers compared to wild-type individuals (raw p-value = 0.04 and Benjamini–Hochberg q-value = 0.20; Table 3). It’s important to note that a difference of 1.00 point represents 21% of the genetic standard deviation for this trait (GSD = 4.77 points).

**Table 3:** Analysis of five performance traits in animals genotyped for the NC_037355.1:g.4039268G > A variant

Trait	N of animal per genotype		B effect (SD)	Raw p-value	BH q-value
	Wild-type	Het			
Birth weight (in kg)	7928	488	− 0.08 (0.13)	0.54	0.76
Ease of calving (score from 1 to 5)	7926	488	0.00 (0.01)	0.91	0.91
MDev at 210 days (score on 100)	4404	271	− 1.00 (0.49)	0.04	0.20
SDev at 210 days (score on 100)	4404	271	0.24 (0.48)	0.61	0.76
Weight at 210 days (in kg)	4146	255	− 0.71 (1.14)	0.53	0.76

MDev: Muscular development. SDev: Skeletal development. N: Number. Het: Heterozygous. B effect: effect size. SD: Standard deviation. P-value: Student's t-test. BH q-value: Benjamini–Hochberg q-value considering all the tests performed

## Discussion

This article presents a powerful approach that allowed us to identify and characterise a novel *GNPAT* deep intronic splicing variant responsible for recessive RCDP in Aubrac cattle. Our strategy's effectiveness is primarily due to the unique structure of the bovine populations and the availability of extensive pedigree, genomic, and phenotypic data collected for selection purposes [5].

Cattle breeds are genetically small populations established around 150 years ago from a limited number of founders. Over the last 50 years, the genetic diversity within these breeds has been further reduced due to the heavy use of influential sires through AI [47, 48]. Typically, the population size of cattle breeds (Ne) ranges from 12 to 150, and the minimum number of ancestors contributing to 50% of the breed's gene pool varies from 5 to 71, as reported in a recent study of 26 cattle breeds reared in France (https://idele.fr/detail-dossier/varume-resultats-2023; accessed 2024/03/28). This low genetic variability within breeds starkly contrasts with the very high genetic variability observed at the species level. This contrast is underscored by the discovery in 2019 of 84 million SNPs and 2.5 million small insertion-deletions (equivalent to one variant every 31 bp) in a collection of 2703 individuals, which represents a significant proportion of global cattle population diversity, as part of the 1000 Bull Genomes Project [49].

As a result, it is feasible to capture most of a breed's gene pool by sequencing only its major ancestors, for whom biological material, often preserved in the form of frozen semen straws, is available even decades after death. Additionally, ancient genetic variability can be captured by repeating this sequencing effort across numerous independent breeds. This context explains why, after performing homozygosity mapping of the RCDP locus within a 1.6-Mb interval on chromosome 28, we were able to reduce the number of candidate variants from 3115 down to a single one: a deep intronic substitution located in the 11th intron of the *GNPAT* gene (NC_037355.1:g.4039268G > A).

Although this type of variant typically does not impact gene expression, it can occasionally lead to severe splicing defects, often resulting in monogenic disorders due to a partial or complete loss of gene function (e.g. [50, 51]). To our knowledge, the only example reported so far in cattle is a SNP in intron 2 of the gene encoding myostatin (MSTN), which causes muscle hypertrophy in the Blonde d'Aquitaine breed [52]. Since several loss-of-function variants in the coding part of *MSTN* have previously been identified in double-muscled cattle [53, 54], the authors took a candidate gene approach. They began by sequencing the cDNA of the gene and then its intronic regions after detecting an abnormal transcript.

In the present study, we identified the NC_037355.1:g.4039268G > A substitution as the only remaining candidate variant after conducting homozygosity mapping and whole genome sequence analysis. We hypothesised that it would alter the correct splicing of *GNPAT* and carried out a complementary series of in vitro, in vivo, and in silico analyses to validate this.

Due to the rapid degradation of RNA at room temperature shortly after death and storage of samples at − 20 °C, we could not analyse *GNPAT* expression in homozygous affected animals. We therefore chose to analyze the splicing of two minigenes, each containing intron 11 with either the ancestral or derived allele of the NC_037355.1:g.4039268G > A substitution, along with the two flanking exons. This analysis was conducted in HEK293T transfected cells. Additionally, we examined *GNPAT* in blood RNA samples from three heterozygous carriers and three wild-type Aubrac cattle (Fig. 5).

Both analyses confirmed that the derived allele was associated with abnormal splicing patterns, likely mediated by an increase in the binding capacity of an SF2/ASF splicing factor encompassing the deep intronic substitution, as predicted by the ESEfinder 3.0 software. The in vitro analysis revealed a transcript with an 86 bp cryptic exon, which was also observed in vivo, along with two other aberrant transcripts resulting from incomplete splicing of the newly formed introns surrounding this cryptic exon.

Although the expression levels of the two minigene constructs were similar in vitro, the aberrant transcripts appeared to be less abundant than the correctly spliced ones in the blood of heterozygous animals. We propose that this difference is attributed to the degradation of the mis-spliced transcripts containing premature termination codons (PTC) through non-sense-mediated mRNA decay (NMD) in vivo but not in vitro. This is because NMD cannot occur in the context of the minigene, where the PTC is positioned in the last exon, unlike in the complete gene sequence in vivo.

The translation of transcripts that include all or part of intron 11 would produce proteins that are missing the last 21% of the amino acids of the bovine GNPAT protein (Fig. 5e). This shortened version would lack the peroxisomal targeting signal 1, which is essential for sorting the majority of peroxisomal proteins to this organelle [55, 56]. Therefore, whether due to NMD, frameshifts, or targeting errors, our findings indicate that the splicing defects caused by the NC_037355.1:g.4039268G > A deep intronic variant will significantly reduce the amount of functional GNPAT protein present in the peroxysomes of homozygous mutants.

The glycerone-phosphate O-acyltransferase, which is encoded by the *GNPAT* gene and is also known as dihydroxyacetone phosphate acyltransferase (DAP-AT, DAPAT and DHAPAT), is an enzyme that is exclusively located in the peroxisomal membrane. This enzyme plays a crucial role in the first step of synthesising ether phospholipids, including plasmalogens [57]. As for other proteins involved in peroxisomal protein import (PEX5 and PEX7) or ether phospholipid synthesis (AGPS and FAR1), variants in the gene encoding GNPAT have been reported to cause RCDP in humans [15–20].

RCDP is a severe developmental disorder resulting from defects in plasmalogen synthesis. Patients with RCDP typically exhibit skeletal dysplasia characterised by rhizomelic shortening of the limbs, punctate epiphyseal calcifications, and distinctive facial features such as a broad nasal bridge, epicanthus, a high-arched palate, micrognathia, and dysplastic external ears. Other clinical features include congenital cataracts, contractures, seizures, severe growth and psychomotor retardation, and a markedly shortened life span [16, 58, 59]. The severity of the syndrome is determined by the degree of plasmalogen deficiency, which is influenced by the specific gene and type of variant involved [60]. For instance, erythrocyte plasmalogen levels are almost undetectable in the classic severe form of RCDP, while they reached up to 43% of average control levels in a study focused on 16 patients with mild RCDP [61].

To our knowledge, no deep intronic variant in the *GNPAT* gene has been reported in humans. However, a literature review identified three variants that, similar to the bovine variant reported here, are predicted to produce NMD-targeted mRNAs and proteins truncated in the C-terminal region (i.e., NM_014236.4:c.1428del, c.1483del, and c.1575del, designated *GNPAT* c.1428delC, c.1483delG, and c.1575delC in [17, 62, 63], and resulting in frameshifts starting at amino acid positions 477, 495, and 525, respectively). In patients homozygous for any of these three deleterious variants, plasmalogen levels were found to be undetectable or nearly zero, suggesting that they do not produce functional GNPAT proteins. Additionally, GNPAT activity and protein were not detected in cultured fibroblasts from patients with the *GNPAT*^c.1428delC/c.1428delC^ and *GNPAT*^c.1483delG/c.1483delG^ genotypes [62, 63].

Due to limitations related to stillbirth and the freezing of necropsied specimens, we could not examine the neuromuscular manifestations or measure plasmalogen levels, which are typically evaluated in red blood cells using gas chromatography/mass spectrometry [64]. Nevertheless, through imaging and skeletal examination of three calves homozygous for the *GNPAT* deep intronic variant, we observed rhizomelic shortening of the limbs and punctate epiphyseal calcification, both hallmarks of the classic severe form of RCDP, along with multiple craniofacial malformations.

Furthermore, by combining large-scale genotyping with analysis of pedigree and performance records available in the French national bovine database, we documented increased levels of juvenile mortality with complete penetrance in the offspring of at-risk versus control matings. A significant effect was observed for the 0–2-day period (+ 5.69%; Benjamini–Hochberg q-value < 1.20e−10), consistent with the stillbirth of the cases referred to the ONAB. A moderate effect was also noted for the 2–14-day period (+ 1.28%), with suggestive or significant q-values (namely 0.10 and 0.3) after Benjamin-Hochberg correction considering either all comparisons, or only those between mating types 1 × 1 and 0 × 0.

Recessive loci with variable expressivity, influenced by modifying genetic or environmental factors are not uncommon in nature, and several examples have been reported in cattle over the past decades (e.g., [6, 65, 66]). Therefore, it would not be surprising to encounter reports of homozygous calves with milder skeletal malformations in the future. This has recently occurred with recessive cleft lip in Limousin cattle due to a frameshift mutation in *MYH3* [67], which was initially described by our team as causing juvenile mortality due to feeding difficulties. A mildly affected heifer, aged 2 years, was subsequently reported by Jacinto et al*.* [68].

In addition, we would like to highlight that, in contrast to the affected calves reported in this article, the clinical features observed in GNPAT-deficient mice do not fully align with those typically reported for human RCDP. Mice that are homozygous for targeted inactivation of the *Gnpat* gene exhibit a complete absence of plasmalogens, male infertility, defects in the eyes and central nervous system development, abnormal behaviours, and mild skeletal abnormalities characterised by disproportionate dwarfism with shortened proximal limbs [69].

Overall, *Gnpat* KO mice are viable. However, about 40% of them die prematurely within the first four to six weeks, while others, especially females, have a normal life expectancy. This discrepancy between humans and mice in the severity of clinical features associated with the inactivation of a gene linked with RCDP was also observed for *Pex7* (reviewed in [70]). In view of these elements, calves homozygous for the NC_037355.1:g.4039268A allele could serve as a reliable large animal model for RCDP. This model presents a promising alternative to mouse models, particularly for investigating how the impairment of plasmalogen biosynthesis may influence the process of endochondral ossification in this condition.

The availability of a probe to genotype the *GNPAT* deep intronic variant on the EuroGMD array will provide a convenient method for managing this locus in selection. It will facilitate the identification of heterozygous carriers among the animals genotyped for genomic evaluation, thus allowing for the avoidance of mating between these carriers. Consequently, no more calves affected by RCDP should be born in the short term.

The efficient identification of heterozygous carriers as part of routine genotyping, combined with advances in reproductive biotechnologies in livestock breeding, also allows for the potential production of limited numbers of case and control individuals in experimental farms. This can facilitate further functional analyses and translational research between cattle and humans. Techniques such as oestrus synchronisation, poly-ovulation, embryo collection, preimplantation diagnosis, embryo freezing and embryo transfer have been successfully employed over the past two decades to study developmental processes, such as horn ontogenesis in bovine fetuses (e.g., [71–73]).

If such an experiment were to be conducted, we would ethically recommend that the case and control embryos be reimplanted into cull cows (cows that have reached the end of their productive careers) and that these embryos be collected from commercial abattoirs during fetal organogenesis, which ends at approximately 70 days (or one quarter) of gestation.

Finally, analysing performance records for thousands of genotyped cattle we report a suggestive reduction of muscular development (MDev) at the age of 210 days in heterozygous carriers versus wild-type inviduals (raw p-value = 0.04 and Benjamini–Hochberg q-value = 0.20; n = 271 and 4404 individuals, respectively). Although, this reduction is not statistically significant after correcting for multiple testing, its magnitude, representing 21% of the genetic standard deviation for this trait, is comparable to that of a QTL with a relatively high effect.

Moreover, a study on *Gnpat* knock-out mice has demonstrated altered development and function of the neuromuscular junction, leading to reduced muscle strength [74]. This finding highlights the need for further investigation in cattle by analysing larger cohorts of animals phenotyped for MDev and conducting functional studies on muscle samples collected after slaughter. These observations in both bovine and mouse models suggest the importance of a detailed phenotypic characterisation of muscle development and function in humans who are heterozygous for deleterious variants of the *GNPAT* gene.

## Conclusions

In conclusion, this study highlights the value of large datasets available in cattle for two primary purposes: (i) detecting causative variants outside the coding regions and (ii) characterising their phenotypic effects. This is exemplified by our report of the first large animal model of RCDP in humans, which is caused by a deep intronic splicing variant of the *GNPAT* gene.

## Supplementary Information

Additional file 1: Table S1. Details of the whole genome sequences used as controls in this study. See the following URLs for more information on the biosample and bioproject IDs: https://www.ncbi.nlm.nih.gov/biosample/ and https://www.ncbi.nlm.nih.gov/bioproject/. Nb_Ind_Breed: Number of individuals per breed.

Additional file 2: Figure S1. Radiographs of an affected calf. Cr: cranial orientation. Scale bar = 10 cm.

Additional file 3: Table S2. List of homozygous positional candidate variants found in the genomes of two RDCP-affected calves. Chr: Chromosome. "Present_in_39_Aubrac_controls" and "Present_in_1828_controls_from_other_breeds" indicate whether the variant was observed in at least one of the 39 Aubrac individuals not carrying the at-risk haplotype or in 1828 genomes of individuals from 70 other breeds used as controls (see Additional file 1: Table S1).

## Data Availability

The WGS data on the RCDP-affected calves are available at the European Nucleotide Archive (www.ebi.ac.uk/ena) under study accession no. PRJEB76441.
