# Supplementary figures and images for: A bovine model of rhizomelic chondrodysplasia punctata caused by a deep intronic splicing variant in the GNPAT gene
Source: Genet Sel Evol. 2025 May 20;57:23. doi: 10.1186/s12711-025-00969-z (PMC12090490; doi:10.1186/s12711-025-00969-z)

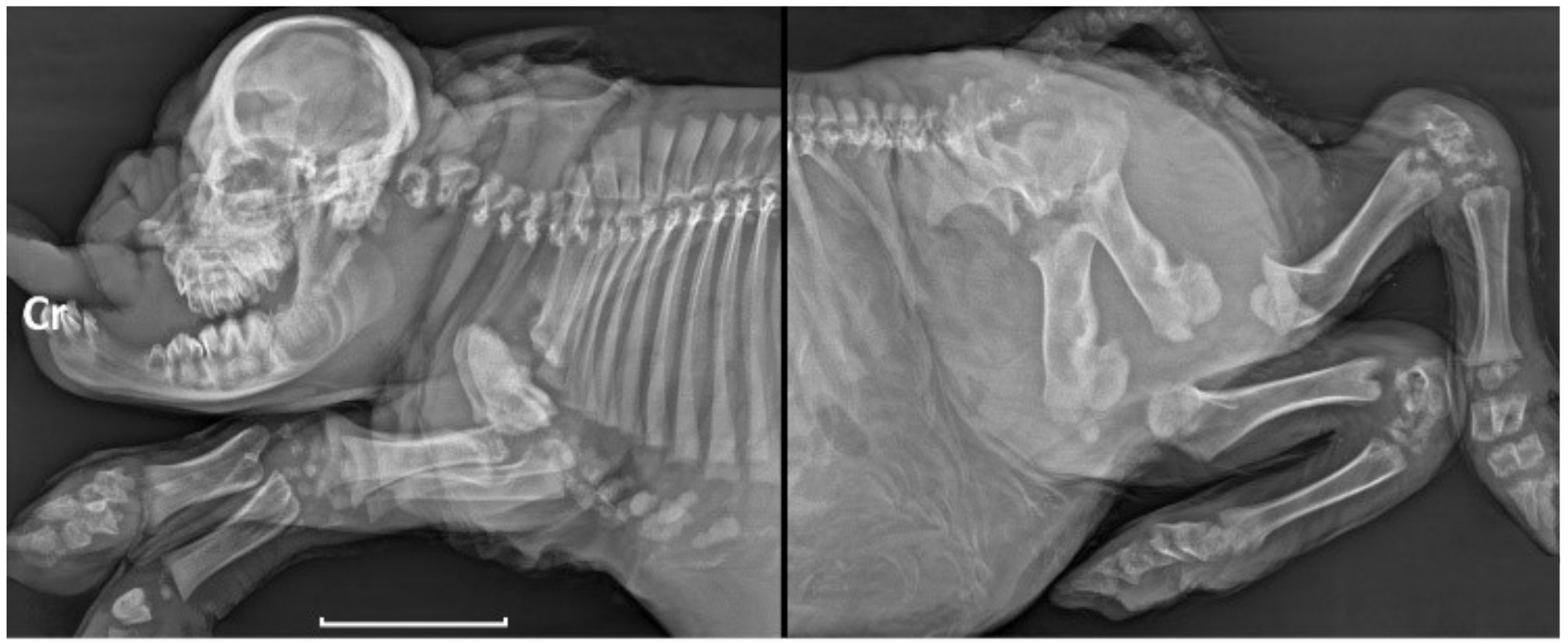

Supplement: Supplementary file 2 — Additional file 2: Figure S1. Radiographs of an affected calf. Cr: cranial orientation. Scale bar = 10 cm. [file 12711_2025_969_MOESM2_ESM.png]
